# Persistence of Use Among Amazigh People of Medicinal Plants Documented by Ibn al-Baytar (Early 13th Century CE)

**DOI:** 10.3390/plants14030342

**Published:** 2025-01-23

**Authors:** Wendy L. Applequist

**Affiliations:** Missouri Botanical Garden, St. Louis, MO 63110, USA; wendy.applequist@mobot.org

**Keywords:** Algeria, Berbers, Jamal Bellakhdar, historical ethnobotany, Islamic medicine, Lucien Leclerc, medicinal plants, Morocco, North Africa

## Abstract

The long-term stability of orally transmitted ethnopharmacopoeias is of interest, but difficult to study for lack of information on plants used by a specific past culture. Similarities between modern Italian ethnopharmacopoeias and Dioscorides’ classical text have been proposed to derive from modern replacement of traditional practices with those from published translations of Dioscorides. Ibn al-Baytar produced the best compendium of medicinal substances in medieval Islamic science. He gave “Berber” common names for some plants, which were presumptively used by North African Amazigh people. Since Amazigh traditional knowledge was largely transmitted orally, with little access to medieval literature, this allows comparison of historic (>750 years ago) practices and modern practices that are unlikely to be causally derived. Presumptive identities for Ibn al-Baytar’s plants with Berber names were obtained from key references. Recent ethnomedicinal publications from Amazigh-populated areas in North Africa were surveyed for reports of those species and homologous common names. Of 46 historically used plants, an estimated 60.9% are still used in Amazigh regions, 78.6% with homologous common names. This is likely to underestimate persistence of species use across the entire local pharmacopoeia. Second, emulating a published analysis of Dioscorides, plants reported by three large recent studies in Amazigh regions were compared with plants recorded by Ibn al-Baytar and in a comprehensive modern Moroccan reference. Between 58.2% and 73.8% of species included in individual studies were recorded by Ibn al-Baytar; of the 46 shared among all three, 82.6% were recorded by Ibn al-Baytar and 100% by the modern reference. An historical compilation may be more likely to mention plants that are widely used today simply because a thorough author could document most plants that were widely used at the time; use of such data to assess causality should take that effect into consideration.

## 1. Introduction

A question of interest in ethnobotany is the degree of change in indigenous medicinal knowledge, which is usually orally transmitted, and practice over long times. It is obvious and uncontroversial that change occurs in any culture, but also that some transmission of useful knowledge between generations is essential to survival. Many cultures or regions have been repeatedly studied by ethnobotanists in the modern era, so that surveys taken a generation or two apart may be compared. Relatively few studies address the more difficult question of how closely a culture’s present body of medical knowledge might resemble knowledge from the much more distant past, centuries or millennia ago. It is increasingly acknowledged that oral traditions can preserve some culturally important knowledge, not necessary for daily survival, for several thousand years or more, e.g., [1,2,3]. How long might people continue to hand down knowledge of medicinal plants? Most medicinal plants are unlikely to be preserved in archaeological sites, and when found, their interpretation is often disputable (even regarding their status as artifacts if they are not in a preserved container [4]). In regions with a long history of indigenous or colonial literacy, a more fruitful approach can be to compare modern ethnobotanical surveys to information from historical literature (including unpublished documents and herbarium specimens).

### 1.1. Studies Utilizing Colonial Records

Certainly one of the most substantial studies to use colonial records is Bussmann and Sharon’s comparison of extensive modern ethnomedicinal data from Peru and Ecuador, with equally substantial data published by the Spanish colonial author Martínez de Compañón in 1780 [5]. Of 526 plants historically used in Peru, 73% were sold in markets or found to be used locally. Conversely, 42% of the species sold in modern markets, which included many post-colonial introductions, had been recorded by Martínez de Compañón. Similarly, Van Andel et al. compared 228–242 Surinamese plant species recorded as useful in Daniel Rolander’s diary, which he kept in 1754–1756, with plants in modern use [6]. About 208 (at least 86%) were still considered useful in Suriname at present, and 54%, especially crop, timber, and ornamental species, had at least one modern use corresponding to a historical use. In Brazil, Alcantara-Rodriguez et al. [7] reported that 80% of the 256 species described as useful in the *Historia Naturalis Brasiliae* (1648) had similar uses recorded in recent literature. This represents a knowledge survival period of ca. 280–360+ years (since the earliest reference Alcantara-Rodriguez et al. used as a source of “recent” traditional knowledge began publication in 1926).

Data from herbarium specimens have also been used to document historical use. Van Andel et al. analyzed the Hermann Herbarium, a bound collection of 51 Surinamese useful plant specimens probably made around 1687 [8]. Forty-eight species were represented, almost all known today as useful; all included medicinal plants were still in use, though commonly for different purposes. Similarly, Soelberg et al. [9] located herbarium specimens from 1695–1697, 1799–1803, and 1817 that recorded medicinal plant uses by the Fante, Ga, and Ashanti people, respectively, all of whom reside in modern Ghana. For all three collections combined, at least 75% of the species were still used, though only 31% of specific uses persisted. In both studies [8,9], 52–59% of common names persisted into modern times. A caveat for both is that small collections of useful plants may disproportionately represent well-known species, whose names and use would be most likely to persist.

Limitations of historical comparative studies include the facts that some plants in older literature will not be identifiable and that ethnobotanical surveys can rarely document all current knowledge. Both would result in underestimation of the proportion of species that persist in use. Studies of this nature, conducted in countries where most local knowledge transmission over the relevant time frame would have been oral, demonstrate that substantial preservation of an ethnopharmacopoeia over a period of at least several generations is common. However, studies of time periods over 200 to 300 years are rare.

### 1.2. Historical Comparisons Involving Dioscorides

Literate scholarship in some regions is of such antiquity as to create the possibility of exploring changes in plant use over much longer periods. The longest time horizon for European studies was established by Leonti et al. [10,11] who examined the proportion of currently used species or genera in various Italian datasets that had been documented in Dioscorides’ *De Materia Medica*, a massive compilation of medicines known in the Roman empire over 1900 years ago. (Of note, a recent study involving some of the same authors [12] similarly compares Dioscorides to selected Central European references from the medieval, Renaissance, and modern eras).

Leonti et al. [10] first compared current medicinal plant lists from Sardinia and Sicily, two Italian islands, with each other and with Dioscorides. Introduced species were excluded from analyses; of the native medicinal species, 170 were used on both islands, while 247 species found on both islands were known to be used only on one. According to plant identifications in Berendes [13], Dioscorides mentioned 62% of the 170 shared species and the genera of another 28%, and reported many of the modern uses for the most-cited 15 species on both islands. He was much less likely to mention the species or genera of plants used on only one of the islands.

The statistical analysis of Leonti et al. [10] rejected the null hypothesis that the 170 plants used on both islands were selected randomly on each “from a pool of 1573 (potential medicinal) species” [10] (p. 265), which is the total number of plant species found on both islands. They concluded that the high proportion of shared medicinal species reflects “common cultural knowledge or selection criteria” [10] (p. 265). More controversially, they suggested that those criteria were neither shared antique tradition nor actual benefits, but a more recent uncritical adoption of plants from printed editions of De Materia Medica: “the medicinal plant knowledge in Sicily and Sardinia is a shallow stereotype of… De Materia Medica” [10] (p. 265).

This interpretation may be questioned. First, the number of species not mentioned by Dioscorides supplies evidence that many modern practices derive from other sources. Second, plants are rarely if ever used at random. In addition to observing benefits directly, users may prescreen plants using characters such as flavor and odor that suggest bioactivity, e.g., [14,15,16]. Likewise, plants that are rare, locally endemic, or inaccessible are less likely to be tested, adopted, then used for long periods. Rarer species might also be less useful, for example, if superior chemical content might help a species to flourish or if past human populations have dispersed useful plants, e.g., [17,18,19]. Many native species would not have been considered suitable for medicinal use. Leonti et al. [10] noted that the 170 shared species could have been shared at random if independently selected from a population of 477 to 539 *medicinal* species (higher than the 417 medicinal species actually present on both islands).

Third, Leonti et al. were surely right to say that writing “revolutionized the transmission of knowledge” [10] (p. 265) and “accentuated the homogenization of medicinal plant use” (p. 266). However, Dioscorides’ creation of a written record of medicines already in use would not have immediately or automatically terminated ongoing oral transmission of folk knowledge of them, and little is known about oral transmission in various time periods. The coexistence of two effective routes of knowledge transmission makes assessment of their relative influence challenging, as suggested by Köhler [20] in a study of the writings of Polish botanist Józef Rostafiński (1850–1928). Rostafiński also came to believe, after observing overlap between Polish folk medicine and the writings of Dioscorides and Pliny the Elder, that 19th-century folk medicines were not survivals from pagan culture. Instead, he concluded that they had been introduced after Christianization in written herbals that influenced first the literate nobility and by further dissemination their servants, then finally rural commoners, whose descendants preserved the introduced practices after the upper classes abandoned them. Köhler observed that Rostafiński had overlooked a possible alternative explanation: that the two bodies of knowledge had a common origin, both descending from the folk knowledge that existed before the scholarly works were written.

In a second paper, Leonti et al. [11] compared the current ethnopharmacopoeia of Campania in southern mainland Italy with those of Sardinia and Sicily and with plants reported by Dioscorides, as copied in a book by the well-known physician and botanist Pietro Andrea Mattioli or Matthioli that also discussed additional species [21]. This work, termed in brief I Discorsi, was first published in Italian in 1544 [22] and was the major pharmacological text in Italian universities until the 18th century. Use categories were recorded separately from Dioscorides’ text and Matthioli’s commentaries. Of 120 native species used medicinally in Campania, Sardinia, and Sicily, all but five were identifiable as Dioscoridean plants, which again was viewed as evidence of Dioscorides’ influence.

Leonti et al. [11] conducted a Bayesian inference-based analysis of use reports for 27 frequently-cited species that concluded that 19.7% of their uses were derived from Matthioli as a causal correlation. This analysis assumed that if species mentioned in a use category by Matthioli are more likely than others to be mentioned in that category today, Matthioli should be treated as a causal influence. Further, the causal effect was considered higher for uncommon plants and uses. An expanded follow-up study [23] analyzed use reports for 87 taxa commonly used in all three Italian regions and sought to detect causal influence of both Dioscorides and a second classical authority, Galen, on modern use. In their model, Dioscorides was estimated to have an average 27.6% (15.1–51.7%) causal effect on plant uses in the three regions, while Galen’s causal effect was estimated at 36.4% (14.3–66.2%). Relative local accessibility of Galen’s and Dioscorides’ work in recent centuries was not discussed.

Such analyses may be vulnerable to bias if many remedies actually work. The finite number of remedies that are most effective for each purpose should be more likely to be adopted, passed on, or rediscovered if once abandoned. Leonti et al. noted that De Materia Medica “generally includes widespread species… and commercialized plant products” [10] (p. 265). Though Dioscorides’ work was magisterial, most of the plant uses he described were not his own discoveries or inventions, but documentations of already-existing professional and folk medical practices in the eastern Mediterranean. If those traditions had discovered a large majority of the indigenous plants that were bioactive and plentiful enough to be worth using by professionals, and Dioscorides’ investigations were thorough, he could have recorded most native genera that multiple modern populations from the region would consider useful, even if his opinions had had no direct influence on theirs at all (that being the extreme case of Köhler’s [21] alternative explanation for shared knowledge).

The opposite question, of how many of the plants recorded by Dioscorides persisted in use to the modern era, is one that cannot be answered fully for two major reasons. First, it is never possible to identify all plants in classical literature. Classical descriptions were often very poor or incomplete, and errors could be introduced by hand-copying. Hence, one purpose of early European herbarium collections was to provide definition for the application of names in classical literature [24]. Folk taxa incorporating multiple species can legitimately be treated as entities with persisting use, but it is sometimes not possible to determine even what genus was described. Second, if the interest is in unbroken transmission of knowledge over long periods, which group(s) are the inheritors of recorded knowledge, within which its “persistence” should be checked for? The plants listed by authors such as Dioscorides were not all used within any single region or culture, and large classical compilations seldom provide adequate information about where plants grew or were used to permit expected descendant cultures to be identified.

The studies by Leonti et al. [10,11] showed that many of the native plants considered useful today in southern European datasets were also recorded by Dioscorides’ great classical compilation. However, their belief that that history was for many species broken by an unspecified, but presumably centuries-long, lacuna rejects the assumption that these species could be said to have a 2000-year history of use. Therefore, to demonstrate persistence of plants’ use in folk medicine over very long periods may require a context in which literate scholarship has existed for several centuries, yet ordinary people in later generations did not place enough value on its products that they could be thought likely to have abandoned traditional knowledge for it.

### 1.3. Ibn al-Baytar’s Treatise on Simples

Information of the kind necessary to demonstrate long-term oral knowledge transmission from a forward-looking perspective, starting from the historical document, may be available for small subsets of plants treated by the great Andalusian physician, pharmacist, and botanist Diya’ al-Din Abu Muhammad Abdullah Ibn Ahmad al-Malaqi, commonly called Ibn al-Baytar, who lived from ca. 1197 to 1248 CE. Born in Malaga, Ibn al-Baytar studied with the famed botanist Abu al-Abbas al-Nabati, then traveled widely in North Africa and the Middle East, gathering information on medicines from practitioners along his route. He is known to have authored 11 works, not all of which survive [25]. One of his two major works, known briefly as the *Jami’ al-mufradat* (Treatise on Simples), was a compilation of text about medicinal plants, animals, and minerals from dozens of classical and medieval Arabic sources, supplemented by personal experiments and observations from his field research. The work included over 2300 alphabetically organized entries; because many substances were given multiple entries for different names and others were unintentionally treated repeatedly, an estimated 1400 substances were included [25].

An authoritative discussion by Bellakhdar [26,27] highlights that Ibn al-Baytar was among the most important authors whose medical works were studied in Islamic universities during the flowering of Islamic science. At that time, all books were hand-copied. Eighty-six handwritten copies of the Treatise have survived, which preserved portions of several earlier Arabic works that would otherwise have been entirely lost to history [25]. Printed copies of the Treatise were published in 1874 and 1992, the former with many errors [25]. There was also a 1990 edition published in Beirut, which may be more scholarly. All are very rare outside their region of origin, though Bellakhdar [27] reports that scholarly practitioners of herbal medicine in modern Morocco often possess copies.

Lucien Leclerc published a scholarly French translation in 1887–1883, omitting most of the lengthy text copied from Dioscorides [28]. Earlier translations into modern European languages had been partial, of dubious quality, or both [25,28]. Leclerc’s translation included annotations discussing his and some previous European scholars’ opinions on the identity of many substances. A facsimile with minor omissions was produced in the late 20th century by the Institut du Monde Arabe (Paris), but is no longer sold. Google Books currently makes all three volumes available [29]. There is still no English translation.

Bellakhdar [26,27] published a comprehensive survey of the modern Moroccan traditional (or folk) pharmacopoeia based on years of field research. As part of his analyses of this very large ethnobotanical dataset, he searched five key medieval Arabic reference works for possible citations of each substance and included notes on such citations. This analysis was similar to Leonti et al.’s [10,11] later backward-looking studies, with more diverse historical sources targeted, but with a time gap less than half as long. The expanded third edition of his work [27] described 759 substances in current use, which included a total of 1118 species or individual non-biological products. Only 31.4% of these were considered not to appear in Ibn al-Baytar’s Treatise, which was the most comprehensive of the five. The 239 substances not mentioned included 14 compounded formulas, 25 later-developed industrial products, and 50 species introduced later from other geographic regions. Of the 576 currently used plant species or species groups, 163 were definitely not mentioned in the Treatise and 15 only dubiously mentioned, leaving 67.4% as presumptively mentioned by Ibn al-Baytar, at least in generic categories [27].

El-Gharbaoui et al. [30] analyzed the overlap between use records of Lamiaceae species in modern eastern Morocco and eastern Andalusia and those from Ibn al-Baytar’s Treatise (referred to there briefly as the Compendium). Fourteen taxa were recorded in current use, with *Thymus* spp. treated as a single taxon as they are used interchangeably. All 14 taxa had been described by Ibn al-Baytar, who recorded 195 uses for them. Ibn al-Baytar had recorded 44% of the 95 specific uses recorded in modern Morocco and 28% of the 257 uses from modern Andalusia.

Based on the reported citation by Ibn al-Baytar of many currently used species investigated in those works and three others, Alami Merrouni & Elachouri [31] have recently argued that “a large part of modern traditional knowledge [in Morocco] originated from ancient Arabic-Islamic medieval medicine” [31] (p. 8). Similarly to Leonti et al. [10,11] but more extreme in interpretation, they described El-Gharbaoui’ et al.’s [30] article as indicating that “43% of plant uses may have [been] acquired straight from Ibn al-Baytar text” [31] (p. 8). Their implication is that all modern uses corresponding to a historical use should be assumed to be derived from historical authors, though later generations may have transmitted this knowledge orally. This ignores the prior existence of the folk knowledge that was merely documented by elite authors. These authors demonstrate an evident bias in favor of crediting the scholars of the “Arabic-Islamic Golden Age” for knowledge generation; their schema for the origins of modern knowledge in Morocco depicts North African and African medicine as being influenced by Arabic-Islamic medicine, but not the reverse.

A very recent publication by Mir et al. [32] provides brief comments on a comparison of Ibn al-Baytar’s work to current knowledge in northeastern Morocco. These authors had recently published an ethnobotanical dataset based on surveys of 1177 people in three provinces, both “Berber” (Amazigh) and Arab [33]. While analyzing this dataset primarily in comparison to other modern Moroccan datasets, they also compared it to a list of 363 taxa believed to have been treated by Ibn al-Baytar and identified as being extant in Morocco. They reported that 221 of 241 taxa mentioned by their interviewees had been cited by Ibn al-Baytar. Those 221 taxa were noted to be “15.78% of Ibn al-Baytar’s simples” [32] (p. 5) [assuming precisely 1400 products were included], but they represented 60.9% of his simples that were believed to be found in Morocco. However, 18% of the 241 taxa mentioned by interviewees had no medicinal use reported, so it was not clear what percentage of relevant simples cited by Ibn al-Baytar were documented as medicines in the investigated region. No further details of methods or results were given.

Names Ibn al-Baytar used or cited included Arabic, classical Greek and Latin, and less often regional (Persian, Berber, or Spanish) vernacular names. Though Ibn al-Baytar mentioned a few substances only briefly without detailing their medicinal uses, his clear intent in writing the Treatise was to document substances of medicinal value (“simples”, or individual medicaments). He was not writing a general botany or zoology text, nor did he seek to provide exhaustive lists of common names for included species. Given the nature and intent of his research, his use of a vernacular name from a regional language provides good reason to believe that people from that region were a source of information on the substance’s use, even if, as was often the case, he did not explicitly say so. He thereby supplied us with some ethnobotanical data for those cultures, which provides, to a limited extent, another means of asking a forward-looking question: how many of a group of species reasonably presumed to be used in the 13th century CE, by a specified culture, are still used today by the culture’s descendants?

### 1.4. The Amazigh People and Medical Traditions

Among these, Ibn al-Baytar recorded “Berber” common names for a few dozen substances, almost all plants, often as the only name known for the substance. “Berber” refers to a group of largely North African ethnicities, tribes, or nations, believed to have derived from at least three separate waves of migration [27], some of whose ancestors have lived in northwestern Africa for up to 15,000 years, despite many later invasions and migrations [34,35,36,37,38]. The umbrella term by which present-day groups reportedly prefer to be known is Amazigh (or Imazighen, a plural), which was adopted in part to assert a collective identity for political reasons, and the preferred collective term for their group of languages is Tamazight [39,40]. Most modern ethnobotanical publications continue to use “Berber” for the people and languages, presumably because of the term’s familiarity from past literature. However, it is a term utilized by outsiders and it may be considered derogatory; though its derivation is disputed, it closely resembles Greek and Arabic words for “barbarian”. In this paper, “Berber” refers only to text in historical works that used that term; when the present-day people or language are discussed, “Amazigh” or “Tamazight” will be used.

The Amazigh have for millennia included large settled agricultural communities, as well as nomadic groups. In Ibn al-Baytar’s time, North Africa was largely Muslim, having long since been conquered by the Arabs [40,41,42], but many Amazigh remained culturally distinct. Today there are many “Arabized” Amazigh who are fluent in Arabic and often identify themselves as “Arab” even if that is not their actual ancestry [42,43]. Conversely, Bellakhdar observes that much Moroccan Arabic culture has been strongly influenced by “berbère” culture, so that the mixture of the two cultures is “indissociable et non réductible” (inseparable and not reducible) in his country [27] (p. 34).

Traditional Amazigh culture reportedly was primarily oral (despite having an indigenous alphabet, Tifinagh, used for inscriptions and short messages [44]), and until recently Amazigh communities tended to have low literacy rates, e.g., [43,45]. Medicinal knowledge in many communities is transmitted mostly by women, who have traditionally had particularly low literacy rates [43]. Fakchich and Elachouri [46] and Ouhaddou et al. [47] reported 32.3% and 52% of their interviewees, respectively, to have had no formal education. Eddouks et al. [48], in their large ethnobotanical survey that included 1616 people from the Tafilalet area, asked interviewees where they obtained knowledge about plants; though 83.4% had at least primary school education, only 6.75% said “reading”, while most indicated “heritage”, learning from others, or personal experience. In an Algerian Amazigh population surveyed by Bendif et al. [49], 73% of those using herbal medicine were women, and 60.27% were illiterate; only 2% of medicinal plant use was reported to derive from “reading”, vs. 89% from “experience of their predecessors”.

Bellakhdar [26,27] considers it certain that there has been an unbroken tradition of oral instruction in healing practices (“une chaine ininterrompue”, an unbroken chain [27], p. 71) in Morocco, formerly parallel to a literate scholarly track, and later completely replacing it so far as transmission of Arabo-Islamic medicine was concerned, with one result being the loss of much theory and doctrine. To be clear, he does not suggest that no rural North African Amazigh knowledge holders in more recent centuries ever possessed or read key medieval scholarly texts, only that their access to them was too limited to be the major influence upon what plants people continued to use. The scholarly information regarding Amazigh ethnobotany in Ibn al-Baytar’s book was derived from traditional knowledge already held by the people among whom he traveled. Had large portions of that knowledge later been abandoned by the Amazigh, their respect for oral tradition and the limited physical accessibility of Ibn al-Baytar’s work (or lesser works of other medieval Arabic authorities) to rural Moroccans make it implausible to assume that that literature would have been sufficiently available, at any time in the past few centuries, to bring about its popular restoration. The importance of these facts is that, if a substantial number of medicines from the “Berber” pharmacopoeia of 750 years ago were found to appear in the Amazigh pharmacopoeia of today, the most plausible explanation for the majority of that similarity would not be abandonment of those plants followed by their relatively recent uncritical adoption from Ibn al-Baytar or some other work of medieval Islamic medicine, but the persistence of indigenous, orally transmitted traditional knowledge for ca. 750 years.

In this paper, plants with Berber common names documented by Ibn al-Baytar will be compared to modern ethnobotanical literature dealing with traditional medicine among Amazigh-descended populations to estimate the proportion of those plants that are still in use today, as well as the proportion that still have similar Tamazight common names. (It seems particularly unlikely that common names would ever be revived from medieval literature, had they dropped out of use and been replaced by others, so homologous common names can be assumed to exist through an unbroken history of use). Additionally, species lists from a selected set of three large ethnobotanical surveys from these populations, and the list of species shared among all three, will be checked for inclusion in Ibn al-Baytar’s work (and that of Bellakhdar, the local modern equivalent) to see whether patterns of greater documentation in historical scholarly literature of plants more widely used today, similar to those found by Leonti et al. [11], may be observed.

## 2. Materials and Methods

### 2.1. Comparison of Historical Berber Data to Modern Amazigh Ethnobotanical Data

Medicinal substances for which Ibn al-Baytar reported Berber names were enumerated, with numbered entries cross-referenced in Leclerc’s French translation [28] as being applicable or partly applicable to each. For some, Bellakhdar [26,27] identified additional entries believed to pertain to the same species or group that were not indicated by Ibn al-Baytar as such; these numbers were not included. Substances that were stated to occur in North Africa were not recorded if Berber names were not mentioned because of the possibility that they were used primarily by Arab populations. Special note was made of a few substances without specified-Berber names that were said to come from Berber lands, but they were not included in the main analysis in order to maintain a consistent criterion. Because modern publications generally deal only with botanical medicines, one fungus, one beetle and one bird were excluded (though one lichen was included). Leclerc noted some Berber names to have been doubtfully recorded or written differently, possibly by copyist error, in different manuscripts. All listed variants of such names were recorded.

Presumptive identifications for Ibn al-Baytar’s names were those proposed by Bellakhdar [26,27], or by Leclerc if Bellakhdar did not mention an historical name. Synonymies were confirmed as needed using major taxonomic databases [50,51]. Bellakhdar [26,27] often identified historical names more precisely than Leclerc and identified and corrected a few misattributions by Leclerc with citations of recent literature. Where Leclerc and Bellakhdar [27] definitely and substantially differed on the identity of a name, both proposed identities were recorded so that modern usage and common names pertaining to both suggested identities could be compared, with the expectation that these would demonstrate more support for Bellakhdar’s identifications. In one case, a differing opinion in a nomenclatural reference by Trabut was also recorded [52].

A large group of recent journal articles presenting ethnobotanical data appearing relevant to Amazigh populations, as well as one book, were surveyed for references to the species or genera with which Bellakhdar (or Leclerc) identified historically used plants. Accessible literature was mostly Moroccan, with a lesser portion Algerian. Most regions of both countries having largely Amazigh populations do also have residents of Arab ethnicity, though as Bellakhdar [27] notes, those cultures have mutually influenced one another. Studies from regions reported to have high Amazigh ethnic populations, especially if they reported common names derived from Tamazight languages, were considered relevant regardless of whether interviewees’ ethnic backgrounds were reported. This survey could not be exhaustive, but it was considered that the large number of accessible recent references would almost certainly include any species having frequent use. In a few cases, when the ethnic background of interviewees was unclear, papers were used as evidence of Tamazight names but not Amazigh use data. Publications providing relevant data included [33,46,47,48,49,53,54,55,56,57,58,59,60,61,62,63,64,65,66,67,68,69,70,71,72,73,74,75].

If no Amazigh region-specific use data were found for a species or species group, major reference works on Moroccan and Algerian ethnobotany [26,27,76] and two recent publications on locally marketed Moroccan medicines [77,78] were checked for national-level citations of the plant’s use, especially associated with Tamazight common names. Bellakhdar [27] occasionally mentioned a specific region, of known Amazigh population, in which he had observed a species to be used. However, it was not counted as Amazigh use if he cited Amazigh regions only as a source of Tamazight common names.

When species or species groups (folk taxa) identified with historically used plants were mentioned in those references, any documented common names that were similar to those in Ibn al-Baytar’s compilation were recorded. If the taxa were not found to be in use or only unrelated common names were seen, the historical Berber common names were searched for without taxonomic restriction in a major compilation of North African common names [52] and in ethnobotanical sources that had provided substantial lists of species with apparently Tamazight common names. A few Berber common names were given by Ibn al-Baytar only in Arabic translation of the meaning, so that possible homologues could not be recognized (at least by this author). If a current common name was located that appeared homologous or similar to a historical name, the plausibility of identifying the historical medicine as the species known by that name was considered.

Common names were recorded exactly as printed in original publications, except that for a few of Ibn al-Baytar’s names from Leclerc, vowel marks were added following his own instructions for correct orthography. Leclerc provided a francophone transliteration of names in the Arabic alphabet (e.g., “ch” was used for ش) and used multiple symbols for some letters (especially غ, a “gh” sound sometimes rendered as “r”), while frequently using “k” for both ك and ق. Recent publications vary substantially in practice, e.g., using a wide variety of diacritical marks. Many of Ibn al-Baytar’s Berber names begin with a double alif (اا), which Leclerc stated to be a definite article in the Berber language; this is generally not seen in recent publications. Likewise, the “t” (ت) with which some historical names began was indicated to be a feminine prefix. Although some modern Tamazight common names are variable in spelling and pronunciation (or are variably recorded), many modern publications reported common names only in the Latin alphabet and may have variably rendered identical names. When spelling of modern common names varies among references, all observed variants were recorded.

Two quantitative questions were asked of the resulting data set: (1) Using the best available identifications, what proportion of plants with Berber common names reported by Ibn al-Baytar appear still to be in use in Amazigh traditional practice, or to be in use regionally? (2) What proportion of the plants in use have modern common names homologous to historical names?

### 2.2. Comparison of Modern Amazigh Ethnobotanical Data to Historical Data

A separate analysis was made to determine what percentage of the species recorded in three selected current ethnobotanical studies involving primarily Amazigh populations, and what percentage of the species that were recorded in all three studies, were mentioned by Ibn al-Baytar, or belonged to species groups or genera that were mentioned. For this analysis, there was no requirement that Ibn al-Baytar should have reported the species to be used by Berbers or even in North Africa. This was intended to be comparable to Leonti et al.’s [10,11] comparison of modern data from multiple Italian regions to the species in Dioscorides’ De Materia Medica, which had similarly been derived from knowledge gleaned over a wide area.

The three datasets chosen for comparison were those of Fakchich and Elachouri [46], Ouhaddou et al. [47], and Teixidor-Toneu et al. [63], which represented three geographically distinct regions of Morocco. Fakchich and Elachouri [46] supplied data from 3151 people in multiple communities of eastern Morocco, reporting 148 medicinal plants. Ouhaddou et al. [47] interviewed 400 people in 13 districts of the southwestern province of Agadir Ida Ou Tanane, reporting 110 medicinal species (including two subspecies of one). Teixidor-Toneu et al. [63] conducted an in-depth study of a single ethnically Ishelhin, Tashelhit-speaking rural commune in the High Atlas mountains (hence, this was an entirely non-Arabized Amazigh group); they reported 151 common names and 159 plants, 153 of them identified at least to genus.

Bellakhdar’s [26,27] assessments of whether each of those taxa were mentioned by Ibn al-Baytar (individually or as part of a species group), not mentioned, doubtfully mentioned, or mentioned only in a generic category or lumped in with a similar species, were recorded, and numbers in each category were tallied. Most of these were extracted from his first edition [26] because it had a complete table of products with specific information; species not included in that work were checked for updates in the third edition [27]. Because preferred nomenclature has changed for some species, the cited online nomenclatural databases [50,51] were used to confirm some synonymies. Where a plant in recent studies was only identified to genus, if multiple species of that genus were reported by Bellakhdar [26,27] to be used interchangeably, it was presumed that the species observed was part of that group.

If a species from the regional datasets was not listed by Bellakhdar as being used in Morocco, a note was made regarding whether other species of the genus were listed in that work, and whether any of those species were recorded as being mentioned by Ibn al-Baytar. Species or genera that were not documented by Bellakhdar, except for exotic taxa that Ibn al-Baytar could not have seen, were looked up in Leclerc [28] to determine whether he associated any of Ibn al-Baytar’s species with those names or well-known 19th century synonyms.

## 3. Results

### 3.1. Ibn al-Baytar’s Berber Names

When entries referring to the same species or folk taxon are combined, Ibn al-Baytar reported Berber common names for 46 plants (Table 1). Leclerc [28] remarked upon “Berber” usage or provided “Berber” names for a few other plants, but his added information was not considered as historical because its sources were unclear and it could have been derived from late-19th-century CE scholarship. Eight of the 46 plants had no meaningful identity supported by either Bellakhdar [27] or Leclerc; one of those (no. 1167, *Afersak*) was a fern but otherwise unknown. None of their common names could be identified with common names in current use in Morocco or Algeria, and none had sufficient descriptive text to identify them as species in use. This result was unsurprising, as Bellakhdar would surely have identified them had that been easily done.

Of the 38 plants with proposed identities, 28 of these species or species groups were reported to be in modern use in Amazigh populations. This count excludes no. 6 (*Aakcheroua*), whose proposed identification, only by Leclerc, was very broad and tentative, so that the association of currently and historically used plants appeared dubious. It also excludes no. 1369 (*Ou’ssâb*), for which Leclerc proposed *Lepidium*; Bellakhdar corrected that identification to *Plumbago*, but though only *Plumbago* has homologous common names, only *Lepidium* has Amazigh-specific references. Contrarily, it includes no. 876 (*Tafîfrâ*), a name used for multiple umbels, for which only Trabut’s [52] proposed identity was seen in an Amazigh reference. An additional five plants were reported to be in use at a national level but not specified to be used by Amazigh populations, one (no. 1369) having homologous common names.

Four plants that were at least marginally identified were not reported to be in current use in the region at all, one (no. 2015, *Tamert ou issoûn*) with a homologous common name. There are a couple of plants for which dubiously related common names may be in use for other genera than those referred to by Ibn al-Baytar. As for the eight unidentifiable plants, whose historical names are no longer in use, it is assumed that all are no longer used by the Amazigh. It is possible that some persist in use under different names, but it would be inappropriate for the purposes of this analysis to assume that. Thus, it is estimated that at a minimum 60.9% (28/46) of the plants for which Ibn al-Baytar reported Berber common names remain in use among the Amazigh; this figure would increase to 63% if no. 1369 were counted.

Of the 28 plants found in Amazigh-related datasets, at least 22 (78.6%) had reported common names that appeared etymologically related to the historical name, although some were rarely used. For example, it appears that modern Amazigh populations usually use the Arabic word *zeitoun* for olives, but variants of the historical name *azemmor* do persist. Some plants (nos. 826 [*Tîglîch* or *Tiglilîch*], 1167 [*Afersak*]) had common names whose homology was uncertain. Considering the 34 species listed in either Amazigh datasets or national references, 24 (70.6%) had homologous common names.

Bellakhdar’s identifications were presumed to be more reliable given his greater firsthand knowledge of the North African flora and ethnobotany. Modern common names often, but not always, provide evidence in support of his interpretations (see Table 1; or, contrarily, might have influenced them). For nos. 2 (*Aâthirîlâl*), 250/1170 (*Ousserghînd* or *Serghent*), 539 (*Tazghet* (تزغت) or *Tarakht*), and 1369/1549 (*Ou’ssâb*), modern names homologous to the historical Berber name were recorded for Bellakhdar’s identifications, but not for Leclerc’s. For no. 398/788 (*Tafghaït*), which the two identified as different but rather similar plants, the same common name of *tafgha* is in use for both species, indicating a folk taxon that may well have commingled the two at the time of Ibn al-Baytar’s writing. For no. 1654/2286 (*Ourdjâlouz*), no homologous common names were found. Finally, Leclerc identified no. 658 (*Fezân* or *Kerân*) as wild artichoke, *Cynara scolymus* L., whereas Bellakhdar identified it as the related *C. humilis* L., though saying that *C. scolymus* was mentioned in the treatment. The only dubiously homologous common names reported were applied to *C. scolymus*, not *C. humilis*.

Of substances that Ibn al-Baytar identified as coming from or used in some portion of North Africa, only five that did not have recorded Berber common names were stated to come specifically from the Berber country or the Berbers. These are the plants (or, in one case, a presumptive lichen) numbered 533, 535, 538, 559, and 1002 (Table 2). Two of those five substances were reported to be used today in Amazigh-related references and two in national references, but none appear in much of the literature. The historical Arabic common names reported for 533 and 535 (*Djouz ez-zendj* and *Djouz es-cherc*), now reported by Bellakhdar to be used for unrelated species, are rather generic geographic names (*jawz* means “nut”, which appears in many common names, and the associated adjectives reference the land of origin) so that a change of meaning would not be unreasonable.

No. 1002 (*Deneb es-seba’*) may be another case in which common names suggest a misidentification by European scholars and Ibn al-Baytar himself. Leclerc [28] suggested it to be a thistle (Asteraceae), possibly a *Carduus*, based on Dioscorides’ Cirsium; Bellakhdar [26,27] provided no identification. The entry included quotations of text from Dioscorides, who supplied a short description, and Abdallah ibn Saleh, who reported medicinal use by Berbers near Fez. Dioscorides’ description is of a spiny plant with stems triangular beneath, leaves similar to bugloss but smaller, whitish but only moderately hairy, and apically borne heads with red appendices. An almost identical Arabic common name, given tolerance for misspelling, is used in modern Algeria for *Dipsacus sylvestris* Huds. (Caprifoliaceae), usually treated as a synonym or subspecies of *D. fullonum* L., fuller’s teasel [52]. *Dipsacus* is prickly on all parts and has ridged stems and often dark pink to purple flowers borne in heads. Bellakhdar [27] recorded no usage of *Carduus* in Morocco and recorded *Dipsacus fullonum* as used only for non-medicinal purposes; he noted its mention by Ibn al-Baytar in several entries, but not 1002. Ibn al-Baytar recorded no personal observations of no. 1002. Given the limitations of historical plant descriptions, it seems plausible that his entry could represent a conflation of Dioscorides’ report of *Carduus* and North African reports of *Dipsacus*.

The three non-plant substances for which Ibn al-Baytar reported Berber common names were truffles (nos. 411 and 1964), cantharides beetles (no. 995), and the flesh of the *saqr* or *yanina* (a small falcon-like bird, no. 1404). Non-plant substances are seldom reported in modern ethnopharmacological papers, but Bellakhdar [26] reported modern use in Morocco of a substantial number, including all of these (if a cited reference to use of falcon skin in magic is counted); he reported homologous Amazigh common names for truffles and falcons.

### 3.2. Comparison of Modern Medicinal Species Lists to Ibn al-Baytar

Table 3 summarizes the appearance or non-appearance of species from three modern Moroccan ethnobotanical datasets, in the opinion of Bellakhdar [26,27], in the work of Ibn al-Baytar. Of Teixidor-Toneu et al.’s [63] 153 identifiable medicinal plants from a single High Atlas Amazigh community, 89 were considered to have been recorded as mentioned by Ibn al-Baytar. These include two plants identified to genus (*Cinnamomum* sp. and *Boswellia* sp.) for which several species can be used interchangeably and one, *Aristolochia paucinervis* Pomel, that probably represents the taxon Bellakhdar [26] called *A. longa* L. An additional six plants were considered to have been mentioned generically or lumped in with related species, and two to have been doubtfully so mentioned. One was considered to have been doubtfully mentioned in its own right, and three plants identified only to genus (*Verbascum* sp., *Juniperus* sp., *Linum* sp.) belong to genera which Ibn al-Baytar mentioned. Of the remaining 52 species, Bellakhdar specified that Ibn al-Baytar had not mentioned 26, while the final 26 were not recorded in Bellakhdar’s work as useful. One of the former is suspected to have been an erroneous identification of an important species, and twelve of the latter had other members of their genera listed, at least in an obsolete taxonomy or in notes.

In Fakchich and Elachouri’s [46] dataset, 148 medicinal plants were reported from eastern Morocco. Nomenclature in this paper was often erroneous. Three names were incomplete or invalid and could not be identified. Of the remaining 145, 107 were considered by Bellakhdar [26] to have been mentioned by Ibn al-Baytar, with two others uncertainly in that category, four to have been included in a generic entry or with related species and three doubtfully so included, and 22 not to have been mentioned. Six were not recorded as useful by Bellakhdar; for four of those, other species of the genus were listed, sometimes several species, which, however, were not explicitly treated by Ibn al-Baytar.

In Ouhaddou et al.’s [47] dataset, 111 medicinal taxa were reported from southwestern Morocco; these included two subspecies of *Euphorbia officinarum* L., which had slightly different reported uses. Of these, 73 were considered by Bellakhdar [26] to have been mentioned by Ibn al-Baytar; five to have been included in a generic entry or with related species and two doubtfully so included, with a possible third dependent upon disagreement regarding taxonomy; and 19 not to have been mentioned. Eleven were not enumerated as useful by Bellakhdar; seven of these had other species of the genus mentioned. One of the others is *Carraluma europaea* (Guss.) N.E. Br. (under *Apteranthes*), to which Bellakhdar reports that a common name for useful species of *Euphorbia* is sometimes applied.

In these three individual datasets, 58.2% to 73.8% of reported plants were considered by Bellakhdar [26] to have been included in the work of Ibn al-Baytar. At most 46 species appear in all three datasets. That number involves the following presuppositions: the *Linum* sp. in Teixidor-Toneu et al. [63] is *Linum usitatissimum* L., the widespread, important species cited in the two other papers; the *Thymus* spp. in Fakchich and Elachouri [46] include *T. willdenowii* Boiss., cited in the two others as *T. pallidus* Coss. ex Batt.; and the *Eucalyptus torquata* Lehmann in Teixidor-Toneu et al. can be equated with the *Eucalyptus* sp. in the other two. In all three cases, homologous common names indicate that they are at least the same folk taxon. Of these, 38 (82.6%) were considered by Bellakhdar to have been mentioned by Ibn al-Baytar as individual species or species groups, two probably to have been included in a generic treatment or lumped into related species, and six (13.0%) not to have been mentioned at all. Of the three species reported as “sp.” and presumptively equated with specific species in other datasets, one was in each of the three categories. If those had been excluded, 37 of 43 (86.0%) would have been mentioned by Ibn al-Baytar, and 5 (11.6%) would have been entirely unmentioned.

## 4. Discussion

### 4.1. Persistence of Historical Species

Probably ca. 61% of the medicinal plants reported by Ibn al-Baytar to have Berber common names are still found to be used in one or more North African regions having ethnically Amazigh populations, and most of those have homologous (but, importantly, not always identical) common names. Had the indigenous knowledge of this region’s people been lost after it was documented by Ibn al-Baytar and other classical authors, including those whose writings he saved from oblivion, few Amazigh traditional knowledge holders in later generations would subsequently have had access to their works, much less used them as an authority superseding local tradition. Therefore, the most parsimonious conclusion is that over half of this group of plants (species or folk taxa) persisted in use among the Amazigh through unbroken oral tradition for some 750 years.

Ibn al-Baytar’s inconsistent reporting of Berber names greatly restricts this analysis. He did not report Berber common names for the large majority of species from potentially relevant regions of North Africa, although many of them must surely have been used by Amazigh people. Modern ethnobotanical surveys in regions of high Amazigh ethnicity almost always find the most important plant family, in terms of number of species used, to be Lamiaceae, commonly with 14–20 species listed, e.g., [30,46,48,54,59,62]. El-Gharbaoui et al. [30] determined that Ibn al-Baytar had recorded 193 uses for 14 taxa (species or genera) of Lamiaceae. Despite this, Ibn al-Baytar did not report a Berber common name for any species of Lamiaceae, though many have modern common names that are of Tamazight origin [27,76].

Those Lamiaceae are all species, or genera of which multiple species may be used interchangeably, that are broadly distributed in the Mediterranean. It is reasonable to presume that if Ibn al-Baytar and his educated audience were familiar with these plants under Arabic, and often Greek or Spanish vernacular names, he saw no reason to document local names that his readers would not need to use to obtain or identify medicines. He would not have reported Berber common names for plants that were widely used outside North Africa, unless he quoted an author who happened to mention the name. Such widely used plants, once adopted, might be less likely to fall out of use because their reputation, commercial availability, and, perhaps, superior efficacy could encourage continued use. If Ibn al-Baytar recorded Berber names mostly for species that were not already known to Mediterranean scholars, this dataset might be skewed towards lesser-known or endemic species whose use might more easily be lost, resulting in an underestimate of the persistence of species.

It is also the case that the number of species recorded to be used in Amazigh regions was dependent upon the number of primary literature sources examined. Several recent publications were seen that provided specific use data for a few species previously only recorded from national-level references. Thus, the percentage of species reported to be persisting would have been meaningfully lower had this work been completed just a few years previously. On the other hand, the fact that a few species were recorded in only one or two of numerous references emphasizes that no community preserves all the knowledge recorded by Ibn al-Baytar, which is not a surprise, since no single community ever possessed all that knowledge; it was amassed from multiple sources. Whether it is important that *some* descendants of the medieval Amazigh still recognize a certain plant as useful, if the vast majority do not, is an open question.

As for those that appear no longer to be used, Soelberg et al. [9] emphasize that the abandonment of a medicinal plant or specific plant use does not necessarily mean that it was concluded to be ineffective. They supply a list of alternative reasons for a plant’s abandonment, including cultural changes (migration, ethnic change, urbanization, changing attitudes towards herbal medicine), reduced availability due to overharvest or habitat loss, increased availability of another medicine perceived as more effective, or reduced occurrence of the disease condition. However, the *persistence* of a plant’s use over several centuries is, they argue, good evidence that it is perceived to be effective.

### 4.2. Documentation of Currently Used Plants in Ibn al-Baytar’s Work

Bellakhdar [27] concluded that 365 of 576 plants then identified as used in Morocco nationwide, including exotics, might be found in Ibn al-Baytar’s Treatise on Simples, or 67.4%. Thus, over two-thirds were already in use 740 years previously. In the present analysis of three recent ethnobotanical datasets from small regions of Morocco [46,47,63], which included a number of recently introduced exotic plants, 58.2% to 73.8% of plants recorded in single studies from small regions had been identified by Bellakhdar with plants treated by Ibn al-Baytar. However, of the 46 plants considered to be shared among all three datasets, 82.6% were identified with plants recorded by Ibn al-Baytar.

These numbers are lower than Leonti et al. [10,11] reported for native plants in Italian datasets. Leonti et al. [11] reported that 115 of 120 native plants found in all three of their selected Italian datasets, or 95.8%, could be associated with Dioscoridean plants. The greater overlap between currently widely-used plants and historically used plants in that study, despite the longer time frame involved, can plausibly be attributed to the past availability of Dioscorides’ (or perhaps Galen’s) work in all three regions and the greater influence of academic scholars and literacy in general, as Leonti et al. propose. However, the substantial shared similarity in this study, where treating the historical literature as causal would not be equally plausible, identifies a “floor” for the level of similarity between widely-used plants from the modern ethnopharmacopoeia and an encyclopedic historical work that might be seen even if the historical work had had little influence on modern traditional knowledge holders. Even within “literate” societies, different cultures may vary greatly in the public’s access to written elite scholarship and the authority they bestow upon it, and both should be taken into account in assessing possible influences on modern folk practices.

In fact, the use of Bellakhdar [26,27] as the primary identifying reference for this analysis provides a second demonstration of this effect. His authoritative compilation of medicinal plant data for Morocco is, for that country, the early-21st-century equivalent of Ibn al-Baytar’s work. Many of his use reports came from his own field studies totaling 809 days in the field between 1969 and 2017 [27]. He obviously did not have a causal effect on the modern uses that he recorded. Contrarily, the fact that the large majority of species in the three large individual modern datasets compared, as well as many other recent publications, are listed in his work is evidence that he did a thorough job of seeking out existing usage. Each of those three datasets contained some fraction of plants that Bellakhdar had not documented. However, not a single species that was reported in all three datasets had been overlooked by Bellakhdar, all of them being recorded already in his 1997 first edition [26]. It is entirely logical that the more widespread a species’ use is, the more likely a researcher is to have the opportunity to document it. The same would have been true for Ibn al-Baytar and, for that matter, Dioscorides.

### 4.3. Application of Common Names

Folk taxa that are defined by their general morphology and/or uses often persist with similar circumscriptions for many centuries. It is not uncommon for folk taxa to represent what a taxonomist would say was not a natural group, including species that are relatively distantly related while excluding more closely related species that have different properties. For example, the historical name “تاغيغشت” (*tāghīghesht*) was tentatively identified by Leclerc with two names in Caryophyllaceae that are synonyms of *Silene vulgaris* (Moench) Garcke, and modern homologues are usually associated with *S. vulgaris* or *S. abietum* Font Quer & Maire, e.g., [47,48,63,77]. However, Benlamdini et al. [59] identified “*tighighechte*” as referring to *Saponaria vaccaria* L. (Caryophyllaceae) [also known as *Gypsophila vaccaria* (L.) Sm. or *Vaccaria hispanica* (L.) Rauschert]. Bellakhdar [27] reported three minor variants of the name as being in current use for either *Silene* or *Saponaria* species and considered that Ibn al-Baytar had also treated them interchangeably. Ibn al-Baytar explicitly attempted, across several different entries, to recognize two distinct types of saponiferous plants, though neither Leclerc nor Bellakhdar seems to have fully respected his opinion on that point. Similarly, Ouarghidi et al. [78] report that “*serghina*” or “*tasserghint*” in commerce may refer both to *Corrigiola telephiifolia* Pourr. and to *Petrorhagia illyrica* (Ard.) P.W. Ball & Heywood, also both Caryophyllaceae.

Thus, in many complex genera, the possibility that the application of the name has changed over time cannot be excluded. It is appropriate to regard modern use as the persistence of the usage, and perhaps name, of a folk taxon, which may or may not be a single taxonomically defined species. These uses may represent simply inaccurate dissemination or inconsistent use of a common name across time and place, much as Americans in different parts of the Midwest use the term “pigweed” for different species of Amaranthaceae s.l. However, they could also reflect a deliberate intention to treat the species as similar, an implicit assertion that they are of similar value. It would be of interest to determine whether such pairs or groups of plants in fact share similar bioactivities that are not shared by excluded congeners.

## 5. Conclusions

Over half of the historical medicinal plants for which Ibn al-Baytar provided Berber common names are used medicinally by modern populations with Amazigh ethnic or cultural backgrounds, and homologous Tamazight common names still exist for a large majority of them. This demonstrates that primarily oral transmission of traditional knowledge is capable of maintaining considerable stability in a pharmacopoeia for over 750 years. Furthermore, plants appearing in multiple recent ethnobotanical datasets were more likely to be included both in Ibn al-Baytar’s work and in Jamal Bellakhdar’s modern work than plants from any single dataset, indicating that plants with widespread current uses are more likely to be found in major historical reference works even if the reference work is unlikely to have been responsible for current use. Plants with a long history of continuous use are particularly likely to demonstrate useful bioactivities and should be prioritized by local scientific communities for clinical trials or other forms of biomedical research.

## Figures and Tables

**Table 1:** Species for which Ibn al-Baytar recorded Berber names, with numbers and Arabic-alphabet and transliterated names from Leclerc [28]; suggested botanical identities, from Bellakhdar [27] unless otherwise noted; use reports for those taxa in studies from Amazigh regions (or, lacking those, in national references); and homologous or similar modern common names, with the species to which they were applied specified as needed. Where Leclerc and Bellakhdar substantially disagreed on the botanical identity of a historical name, both are listed on separate lines. IAB = Ibn al-Baytar.

Number(s) [Leclerc]	Historical Berber Name(s) [Leclerc]	Suggested Identity	Species Reported in Amazigh Traditional Medicine	Homologous or Similar Modern Common Name(s)
2	*Aâthirîlâl* (أاطِرِيلال)	Leclerc: *Ptychotis verticillata* Duby [=*Ammoides pusilla* (Brot.) Breistr.] (Apiaceae)	*P. verticillata* [=*A. pusilla*] [46]; *A. pusilla* [71]	
		Bellakhdar: *Ammi majus* L. (Apiaceae) (confused with *Ptychotis* since historic times; see also [6])	*A. majus* [33,70]	Aatrilal [70]; Āṭrīlāl (آطرِيلال), Ṭrīlān (طرِيلان), Ṭlīlān (طلِيلان) [abbreviation for âṭar îlâl, bird’s foot] [27]
3, 542	*Aacoutsâr* (ااكُثار)	*Bunium* spp. (Apiaceae)	*B. bulbocastanum* L. [63]; *B. mauritanicum* (Boiss. & Reut.) Batt. [=*B. fontanesii* (Pers.) Maire] [60]	*B. fontanesii*, *B. incrassatum* (Boiss.) Amo [=*B. ferulaceum* Sm.?]: Ākut̠ār (آكثار), Akt̠īr (اكثير) [27]
4, 1607 in part	*Aarr‘îs* or *Arghîs* (اارِغيس)	*Berberis hispanica* Boiss. & Reut. [=*B. vulgaris* L. subsp. *australis* (Boiss.) Heywood]	*B. vulgaris* [60,70], *B. hispanica* [46]	*B. vulgaris*: Ârgîs [70], Elghris [58]. *B. hispanica*: ٲغريس [46], Ārġīs (ٲرغيس) [27]
5	*Aamilîlis* (اامِلِيلِس)	*Rhamnus alaternus* L. (Rhamnaceae)	*R. alaternus* [49,55,59,60,65,72,74,75]	M’liles [49]; Melilez [59]; Amliles [53]; Mlilles [71]; Amlilis [72]; Āmlīles (آمليلس), Mlīles (مليلس) [27], Mlilès, M’liless [55], Mlilis [74], Imliles [75]
6	*Aakcheroua* (ااقشروا); also read ااقشزواو and ااقشرون	Leclerc: Chlora? Centaurée? [*Centaurium* or *Blackstonia* (includes *Chlora*) (Gentianaceae)?]	*Centaurium erythraea* Rafn [46,61,62,74,75]; *C. umbellatum* (Gilib.) Beck [55]; *Blackstonia grandiflora* (Viv.) Maire [75]. In Morocco, *C. spicatum* (L.) Fritsch [53]	*Chlora grandiflora* Viv. [≡*Blackstonia grandiflora*: Aqchera [52]
27, 86, 741	*Addâd* (أَدَّاد)—white chameleon (see 742)	*Atractylis gummifera* L. [≡*Carlina gummifera* (L.) Less.] (Asteraceae)	*C./A. gummifera* [46,47,55,57,59,63,72,74] *C. acaulis* [48]	*C./A. gummifera*: Addad [47,57,72,74]; Dâd, Dlagh [59]; Dad [70]; اداد [46]; Leddad [71]; Āddād (آّدّاد), Ddād (دّاد) [27]; Adad [55] *C. acaulis*: الداد [48]
28, 440, 2321	*Adrîs* (ادريس), *Adriâs* (ادرياس)	*Thapsia transtagana* Brot. [=*T. garganica* L. subsp. *decussata* (Lag.) Maire] and *T. villosa* L. (Apiaceae)	*T. transtagana* [57]; *T. villosa* and *T. garganica* L. [incl. *T. decussata* Lag., = *T. transtagana*] [55]; *T. garganica* [61,72,74,75]	Wrak darrias [57]; Daryass [18]; Deryass [77]; Deryās (درياس), Āderyās (آدرياس) [27]; Aderys, Derias [55]; Adharyis [61]; Adaryes [74]; Aderiass [72]
35 [also see 2031]	Sheep’s ear (اذان الشاة), gazelle’s ear (الغزال اذان), [Arabic translations of Berber names]	*Cynoglossum officinale* L. (Boraginaceae)	In Morocco, *C. officinale* [27]	
56, 1145, 2041	*Ardjân* (ارجان), *Argân* (ارقان)	*Argania spinosa* (L.) Skeels (Sapotaceae)	*A. spinosa* [57,62,64,66,73]	Argan [53,57,64,66]; Argane [62]; Argan, ⴰⵕⴳⵯⴰⵏ/آركان [73]; Ārgān (آركان) [27]
61bis, 717	*Azourd* (ازورد) or *Azrour* (ازرور)	*Melilotus* spp. (Fabaceae)	*M. officinalis* (L.) Pall. subsp. *alba* (Medik.) H. Ohashi & Tateishi [48]. In Morocco, *Melilotus* spp. [27]	*M. officinalis*: ازرود [48]. *Melilotus* spp.: Āzrūd (آزرود), Āzūrd (آزورد), Zrūda (زرود) [27]
250, 1170	*Ousserghînd* (اوسرغيند) or *Serghent* (سرغنت)	Leclerc: *Telephium imperati* L. (Caryophyllaceae)	*Telephium imperati* [46]	
		Bellakhdar: *Corrigiola telephiifolia* Pourr. (Caryophyllaceae)	*C. telephiifolia* [46,48,62]	سرغينة [46,48], تاوسرغينت [48], Sarġīna (سرغين), Tasserġint (تسّرغنت) [27]; Serghine, Sarghina [62]; صرغينة (Sarġîna) [54], Serghina [53,77], Tasserghint [78]
260, 390?	*Abou-immoût* (“the father is dead”) (ابويمّوت), *Boukichrem* (بُوقِشرم)	Unidentified; two species described		
280, 416, 442, 1891	*Techtiouân* (تشتيوان)	*Polypodium vulgare* L. (Polypodiaceae)	*P. vulgare* [27 (Azrou)]	Astīwān (استيوان), Aštīwān (اشتيوان), Taštīwān (تشتيوان) [27]; possibly Achtouane, Chtioual, Nechnaouane [76]
375	*Akankan* (اقنقن)	*Verbascum* spp. (Scrophulariaceae)	*V. sinuatum* [33,61,74]; *Verbascum* sp. [63]. In Morocco, 4 spp. [27], Amazigh names given	
398, 788	*Tafghaït* (تافغيت)	Leclerc: *Cynara acaulis* L. [≡*Rhaponticum acaule* (L.) DC. or *Leuzea acaulis* (L.) Holub] (Asteraceae)	In Morocco, *R. acaule* [78]	Tafgha [78]
		Bellakhdar: *Centaurea chamaerhaponticum* Ball [=*Centaurea acaulis* L.] (Asteraceae)	In Morocco, *C. chamaerhaponticum* [=*C. acaulis*] [27,77]	Tāfġā (تافغا) [27], Tafgha [77]
399, 1673	*Tacout* (تاكوت) or *Tanqout* (تانقوت; see note 2296)	*Euphorbia resinifera* O. Berg, *E. echinus* Hook.f. & Coss. [≡*E. officinarum* L. subsp. *echinus* (Hook.f. & Coss.)] Vindt., and *E. beaumieriana* Hook.f. & Coss. [≡*E. officinarum* var. *beaumieriana* (Hook.f. & Coss.) Maire]]	*E. officinarum* subspp. *officinarum* and *echinus* [47,64]; *E. resinifera* [46,66]; *E.* spp. including *E. echinus* [65]; *Euphorbia*, 5 spp. [57]	*E. beaumierana*: Tikiūt or Tikiwt (تكيوت) [27]. *E. officinarum* subsp. *echinus*: Tikiout [57]. *E. resinifera*: تكيوت or تكاوت [46]. Tikhiout [64]. *E. officinarum*: Tikiout [47]
400, 1507	*Taghendest* (تاغندست), *Tîkendest* (تيقندست)	*Anacyclus pyrethrum* (L.) Lag. and *A. depressus* Ball [=*A. pyrethrum*] (Asteraceae)	*A. pyrethrum* [2,46,48,59,60]	Tiguentest [59]; تكنطيس [46]; تاقنديشت [48]; Tiguendizt [77]; Tigenṭast (تكنطست), Īgenṭas (إكنطس), Tigenthast [72], Genṭūs (كنطوس), Taġendest (تغندست) [27]
401, 2185	*Tamchaourt* (تامشاورت) (or *tamsaourt,* تامساورت)	Leclerc: *Meum*, possibly *M. athamanticum* Jacq. (Apiaceae)	In Morocco, *M. athamanticum* [77]	
402, 698	*Tassemmoumt* (تاسمّمْت) (read otherwise by error; feminine of *semmoum* or *assemmam*)	*Rumex* spp. (Polygonaceae)	*R. acetosa* L. [33,47,60,62,72]; *R. conglomeratus* Murray [61,74,75]; *R. vesicarius* L. [33,57]; *R. bucephalophorus* L. subsp. *gallicus* (Steinh.) Rech. [55]; *R. pulcher* L. [33]	*R. acetosa*: Tismoumine [47], Semmamout [72]. *R. vesicarius*: (?) Basmom, Wasmim [57]. *R. buecephalophorus*: Tasemumt, Tasmumt [55]. *R. conglomeratus*: Tassemumt [74], Thassemoumth Guezgaren [61], Tassemumt n yezgaren [75]. Group of 9 species: Tassemūmt (تسمّمت), Tassamam (تسّمم), Tissemumīn (تسّممين), Smamun (سممن), Bassemūm (بسّموم) [27]
413, 1448	*Terhelân* (ترهلان), *Tarhelâ* (ترهلا)	*Inula viscosa* (L.) Aiton [≡*Dittrichia viscosa* (L.) Greuter] (Asteraceae)	*I./D. viscosa* [33,46,48,49,55,61,66,72,73,74,75]	Tinirine, Terrhla [66], Terreklān (Tahraoui et al.), تارهلا [48], Terhalā, Terahlā (ترهلا) [27]
423, 2255 [323,329]	*Tifâf* (تِفاف)	*Sonchus* spp. (Asteraceae)	*S. oleraceus* (L.) L. [55,61,72,74,75]; *S. tenerrimus* L. [73]; *S. asper* (L.) Hill, *S. tenerrimus*? [68].	*S. oleraceus:* Tifaf [53], Tifef [55], Thifaf [74,75], Thiffaf [61], Tilfef [72]. *S. tenerrimus*: ثيفاف [73] Group of 4 spp: Tifāf (تفاف), Tilfāf (تلفاف) [27]
539	*Tazr’et* (تزغت) [sic] or *Tarakht* (تارخت); *Thamah* (طمح) or in 1 ms. *Thalah* (طلج) [sic]; includes 2 types	Leclerc: Sorbier? [*Sorbus* (Rosaceae)]	In Morocco, *Sorbus aria* (L.) Crantz [53]	
		Bellakhdar: IAB’s *tazaġt* is *Rhus pentaphylla* (Jacq.) Desf. [≡*Searsia pentaphylla* (Jacq.) F.A. Barkley] (Anacardiaceae); IAB’s *judar* is *R. tripartita* (Ucria) Grande [≡*Searsia tripartita* (Ucria) Moffett]	*R. pentaphylla* [33,47] *R./S. tripartita* [46,56,69,72]	*R. pentaphylla*: Tazaġt (تزغات [sic]) (plural Tizġā, تزغا), Tazâdt تزاضت), etc. [27]; Tizrah, Tazrah (تيزغة) [52]; Tazzad, Azad [47]; Tizga (تزكا), Azad (أزاد), Tazadt (تازادت) [33]. *R. tripartita*: تيزغة, تيزغت [46].
658	*Fezân* (فزان) or *Kerân* (قران)?	Leclerc: Wild artichoke (Dioscorides citation *Cynara scolymus* L., Asteraceae)	*C. scolymus* [33,46,47,73,74]	Possibly القرني [46], El-Qorni’ (القرنيع) [33] (??)
		Bellakhdar: *Cynara humilis* L., with *C. scolymus* mentioned in same treatment	*C. humilis* L. [62]	
742	*Addâd* (أَدَّاد)—black chameleon, *Ouahîd* (الوهيد) [see no. 27]	Leclerc: *Carthamus corymbosus* L. [≡*Cardopatium corymbosum* (L.) Pers.]		
824	*[al-]Amîroun* (الاميرون)	Leclerc: *Chondrilla* sp. (Asteraceae)		
826	*Tîglîch* (تيقليش) or *Tiglilîch*	*Asphodelus ramosus* L., *A. microcarpus* Salzm. & Viv. (Asphodelaceae)	*A. microcarpus* [53,55]; *A. ramosus* [47,67,74,75]	Possibly Āgellūs (آكلّوس) [27] (??)
842	*Aroûzi* (اروزى)	Leclerc: “Aspalathe” of Dioscorides: species of 3 genera of Fabaceae have been suggested		Unrelated species with somewhat similar names: *Warionia saharae* Benthem ex Benth. & Coss. (Asteraceae): Arouzzir [52]. *Coriaria myrtifolia* L. (Coriariaceae): Arouz [52], Ārwāz (ارواز) [27]
876, 1191	*Tafîfrâ* (تفيفرا or تافيفرا)	Bellakhdar: *Heracleum sphondylium* L. (Apiaceae) possibly due to IAB’s confusion [27] Trabut: *Magydaris panacina* DC. [=*M. panacifolia* (Vahl) Lange] [52]	*Magydaris panacifolia* and *M. pastinacea* (Lam.) Paol. [33]	*M. panacina*, *M. tomentosa* (Desf.) W.D.J. Koch ex DC. [=*M. pastinacea*] and *Athamanta sicula* L.: Tafifra [52], Fafra (فافرة) [33]. Umbels including *H. sphondylium*, *M. panacifolia*, and *M. pastinacea*: Frīfra (فريفر), Fīfra (فيفر), Tafrīfra (تفريفر) [27]
1140	*Azemmor* (ازمّور)	*Olea europaea* L. (Oleaceae)	*O. europaea* [33,46,47,55,57,61,62,63,64,66,69,73,74,75]	Azmour [63,64], Azemour [74,75], Azemmour [47], Azemur [55], Āzemmur (آزمّر) [27]
1167	*Afersak* (افرسق)	*Pteridium aquilinum* (L.) Kuhn (Dennstaedtiaceae), treated with *Dryopteris filix-mas* (L.) Schott (Dryopteridaceae)	*P. aquilinum* [27,55,61,75], as *Pteris aquilina* L. [33]	Possibly Afersīn (افرسين) and similar names [27] (??)
1179	*Tar’îr’ats* [sic] (تارغيرغث), *Kerrout* (كروت), *Tarhîrhecht* [sic] (تاغيغشت)	Leclerc: Struthion, perhaps *Cucubalus behen* L. or *Silene inflata* Sm. [both = *S. vulgaris* (Moench) Garcke] (Caryophyllaceae) Bellakhdar: *Silene* and *Saponaria* spp. (IAB cited by name, not number)	*Sil. vulgaris* [47,63,73,74,75]; *Sil. cucubalus* Wibel (=*Sil. vulgaris*) [55]; *Sil. abietum* Font Quer & Maire [48]; *Sap. vaccaria* L. [59]	*Sil. vulgaris*: Tigheghcht [77], Tighercht [47], Taghighasht [63], Tighighach [74], Tighighache [55], Thaghighachth [75], ⵜⵔⵉⵕⴰⵛⵜ/ثغيغشت [73]. *Sil. abietum*: تغيغشت [48]. *Sap. vaccaria*: Tigheghechte [59]. *Silene* and *Saponaria* spp.: Tīġīġest (تغيغست), Tīġīġešt (تغيغشت), Tāġīġešt (تاغيغشت) [27]
1369, 1549	*Ou’ssâb* or *O’ssâb* (عُصَّاب)	Leclerc: *Lepidium*; *L. latifolium* L.? (Brassicaceae)	*L. latifolium* [74]; *L. sativum* L. (equated with *L. latifolium*) [55]; *L. sativum* [46,73]	
		Bellakhdar: *Plumbago europaea* L. (Plumbaginaceae), which is confused with *Lepidium*	In Morocco, *P. europaea* [27]	‘Uṣṣāb (عصّاب), La‘ṣṣāb (الاعصّاب), La‘ṣṣābat (الاعصّابت) [27]
1604	*Esfaghîgher*? (اصفغيغر) or perhaps *Esghîfer* (اصغيفر)	Leclerc: Bois de serpent; unidentified, dubiously *Ophioxylon* (Apocynaceae)		
1654, 2286	*Ourdjâlouz* (ورجالوز), variant *Ourdjâlour* [ورجالور]	*Bryonia dioica* Jacq. [≡*B. cretica* subsp. *dioica* (Jacq.) Tutin] (Cucurbitaceae)	*B. dioica* [55]	
1655	*Mimoun* (ميمون)	Leclerc: Taminier; *Bryonia alba* L.? (Cucurbitaceae)		
1698	*Harmi* (حرمى) or possibly *Harri* (حرى)	*Xylopia aethiopica* (Dunal) A. Rich. (Annonaceae)	In Morocco, *X. aethiopica* [27]	
1717	*Amz*? (امز)	Leclerc: Spondylium? Unidentified		
1878	*Tâzghallât* (تازغللات or تازغلت)	*Ranunculus* spp. (Ranunculaceae)	In Morocco, 5 spp. of *Ranunculus* [27]	
1902, 2304	*Yakhsis* (يخصص)	*Smyrnium olusatrum* L. (Apiaceae)	*S. olusatrum* [48,61]	Īġsās (إغسس), Īḫses (إخسس), Yaḫsīs (يخسيس), Taḫsīs (تخسيس) [27]
2015	*Tamert ou issoûn* (probably “ogre’s beard) (تمارت ويسون)	Leclerc: *Asplenium trichomanes* L. (Aspleniaceae)		Tamart ou içoun [52]
2047	*Irna* (ايرنى)	Several *Arum* and *Arisarum* spp. (Araceae)	*Arisarum vulgare* O.Targ.Tozz. [33,55,61,71,73]; *Arum maculatum* L. [33,54]; *Arum italicum* Mill. [33,55,74,75]	*Arisarum vulgare*: إرني [73] *Arum maculatum*: Ayerna [54]. Multiple species of *Arum* and *Arisarum*: Īrnī (إرني), Ārnī (آرني), Yernī (يرني), Āyernī (آيرني) [27,33]
2106	*Choûka Meghîla* (مغيلة شوكة) [Arabic translation of Berber name]	*Centaurea calcitrapa* L.? (Asteraceae); not *C. pungens* Pomel unless conflated with *C. calcitrapa*.	*C. calcitrapa* [74]; *C. pungens* [67,69]; in Morocco, *C. calcitrapa* and *C. maroccana* Ball [27]	
2188	*Asmâmin* (اسمامِن) or *Esmâqen* (اسماقن)	Unidentified; the former name is used in the *‘Umdat aṭ-ṭabīb* for *Rumex* spp., but the description does not match [27]		
2206	*Hormi* (حرمى) or *Aghroum* (اغروم) [means “bread”]	Unidentified; Leclerc: possibly = no. 1698?		
2293	*Ouathmou* (واطموا)	Unidentified		
2297 note	*A’cheba kîry* [عشبة قيرى] [prob. Arabic translation], possibly *Ouendjehek* or *Ouendjehel* (ونجهك or ونجهل)	Unidentified		

**Table 2:** Species directly associated by Ibn al-Baytar (IAB) with the Berbers, but without Berber names.

IAB #	Classical Name, Ibn al-Baytar’s Link to Berber People [28]	Suggested Identity	Current Species Used	Similar Modern Common Name(s)
533	*Djouz ez-zendj* (جوز الزنج); it is brought from the desert of the Berbers’ country.	Leclerc: *Sterculia acuminata* P. Beauv. [≡*Cola acuminata* (P. Beauv.) Schott & Endl.] (Malvaceae) [28]		Jawzat az-zenj (جوزة الزنج) for *Xylopia* spp. (see no. 1698), also by some for *Aframomum melegueta* (Roscoe) K. Schum [27]
535	*Djouz es-cherc* (جوز الشرك); it is brought from the Berbers’ country.	Leclerc: *Amomum grana paradisi* L. [=*Aframomum* sp.] (Zingiberaceae) [28]	In Morocco and Algeria, *Af. melegueta* (Roscoe) K. Schum. [27,52]. Bellakhdar: not mentioned by ibn al-Baytar [27]	Jawzat aš-širk (جوزة الشرك) for *Xylopia* spp. (see no. 1698) [27]
538	*Djouz djondom* (جوز جندم); the *Livre des Talismans* says that of the Berbers is the best and strongest.	Bellakhdar: *Lecanora esculenta* (Pall.) Eversmann (lichen) [27]	In Morocco and Algeria, *L. esculenta* [27,52]	
559	*Habb ez-zelm* (حب الزلم); it is brought from the Berbers’ country.	Bellakhdar: *Cyperus esculentus* L. (Cyperaceae) [27]	*C. esculentus* [33,73]	Ḥabb ez-zalam (حبّ الزلم) [27], Habb ez zelim (حب الزليم) [52]
1002	*Deneb es-seba’* (ذنب السبع); Abdallah ibn Saleh saw it used by Berbers near Fez.	Leclerc: “Cirsium. P.”; prior authors suggested *Carduus tenuiflorus* Curtis (Asteraceae) [28]	*Cirsium chrysacanthum* (Ball) Jahand [63]	*C. chrysacanthum*: None similar *Dipsacus sylvestris* Huds. [possibly = *D. fullonum* L.] (Caprifoliaceae): Denb es sebaa (دنب الصبع) [52]

**Table 3:** Numbers of identifiable taxa reported in three recent Moroccan datasets [46,47,63]; the subgroup of species found in all three datasets considered by Bellakhdar [26,27] to have been mentioned by Ibn al-Baytar under individual species or species groups, possibly mentioned, at least possibly included in a generic treatment or lumped with other related species, or not mentioned, or not recorded as useful by Bellakhdar.

	Fakchich and Elachouri [46]	Ouhaddou et al. [47]	Teixidor-Toneu et al. [63]	Shared Species
Total # taxa	145	111	153	46
Mentioned	107 (73.8%)	73 (65.8%)	89 (58.2%)	38 (82.6%)
Possible mention	2 (1.4%)		4 (2.6%)	
Generic mention or possible mention	8 (5.5%)	8 (7.2%)	8 (5.2%)	2 (4.3%)
Not mentioned	22 (15.2%)	19 (17.1%)	26 (17.0%)	6 (13.0%)
Not listed in Bellakhdar	6 (4.1%)	11 (9.9%)	26 (17.0%)	

## Data Availability

No new data were created or analyzed in this study. Data sharing is not applicable to this article.
